# Suppression of αvβ6 Integrin Expression by Polymicrobial Oral Biofilms in Gingival Epithelial Cells

**DOI:** 10.1038/s41598-017-03619-7

**Published:** 2017-06-30

**Authors:** Jiarui Bi, Leeni Koivisto, Aihui Pang, Ming Li, Guoqiao Jiang, Saljae Aurora, Zhejun Wang, Gethin R. Owen, Jiayin Dai, Ya Shen, Daniel Grenier, Markus Haapasalo, Lari Häkkinen, Hannu Larjava

**Affiliations:** 1 Faculty of Dentistry, Department of Oral Biological and Medical Sciences, University of British Columbia, Vancouver, BC V6T 1Z3 Canada; 2 Department of Stomatology, The Fourth Affiliated Hospital, Harbin Medical University, Harbin, 150001 China; 3 Faculté de Médecine Dentaire, Université Laval, Quebec City, QC G1V 0A6 Canada; 4 Center of Stomatology, Tongji Hospital, Tongji Medical College, Huazhong University of Science and Technology, Wuhan, Hubei China

## Abstract

Periodontal diseases manifest by the formation of deep pockets between the gingiva and teeth where multispecies bacterial biofilms flourish, causing inflammation and bone loss. Epithelial cell receptor αvβ6 integrin that regulates inflammation by activating the anti-inflammatory cytokine transforming growth factor-β1, is highly expressed in healthy junctional epithelium that connects the gingiva to the tooth enamel. However, its expression is attenuated in human periodontal disease. Moreover, *Itgb6* ^−/−^ mice display increased periodontal inflammation compared to wild-type mice. We hypothesized that bacterial biofilms present in the periodontal pockets suppress αvβ6 integrin levels in periodontal disease and that this change aggravates inflammation. To this end, we generated three-week-old multi-species oral biofilms *in vitro* and treated cultured gingival epithelial cells (GECs) with their extracts. The biofilm extracts caused suppression of β6 integrin expression and upregulation of pro-inflammatory cytokines, including interleukin-1β and -6. Furthermore, GECs with β6 integrin siRNA knockdown showed increased interleukin-1β expression, indicating that αvβ6 integrin-deficiency is associated with pro-inflammatory cytokine responsiveness. FSL-1, a synthetic bacterial lipopeptide, also suppressed β6 integrin expression in GECs. Therefore, biofilm components, including lipopeptides, may downregulate αvβ6 integrin expression in the pocket epithelium and thus promote epithelial cell-driven pro-inflammatory response in periodontal disease.

## Introduction

About half of the adult population of the United States suffers from periodontal diseases, in which bacterial biofilm induces gingival inflammation, leading to tissue degradation, alveolar bone loss around teeth and even tooth loss^1^. These chronic inflammatory processes have been shown to be potential contributing factors to many systemic conditions, including cardiovascular disease, cerebrovascular diseases, rheumatoid arthritis, Alzheimer’s disease and cancer^2^.

Healthy junctional epithelium (JE) connects gingiva to tooth enamel, and this seal prevents bacteria from entering the gingival tissue^3^. When periodontal disease develops, JE moves from the enamel and transforms to pocket epithelium (PE), and a periodontal pocket forms. Bacterial biofilms begin to flourish in these pockets between the tooth and PE. The bacteria can invade PE cells and sometimes also the connective tissue, but this invasion is generally quite limited, considering the abundance of biofilm bacteria in the deep periodontal pockets (10^6^–10^9^ per site)^4^. Therefore, the role of JE/PE, which separates the bacteria from the connective tissue and the inflammatory infiltrate, is thought to be essential for protecting the periodontal tissues^3^. However, the mechanism of this protection in the regulation of periodontal inflammation is still unclear.

*In vitro* studies investigating the response of the gingival epithelial cells (GECs) to dental plaque microorganisms have shown that these cells respond to most subgingival bacterial species by increasing their pro-inflammatory cytokine expression^5–7^. These cytokines include, but are not limited to, interleukin-1β (IL-1β) and IL-6. Thus, the dental plaque bacteria-induced release of pro-inflammatory cytokines from the JE/PE could play a central and initiating role in the pathogenesis of periodontal disease. However, little is currently known about the molecular pathways in the host JE/PE cells that regulate their cytokine response to complex, mature bacterial biofilms.

Transforming growth factor-β1 (TGF-β1) and IL-10 are considered the major balancing anti-inflammatory cytokines that, in the periodontal disease process, counter the effects of pro-inflammatory cytokines in the inflammatory infiltrate^8^. The important anti-inflammatory function of TGF-β1 has been evidenced in studies with TGF-β1 knockout animals, which die soon after birth from massive infiltration of lymphocytes and macrophages into many organs^9, 10^. The surveillance role of TGF-β1 provides an innate immunosuppressive action on T-cells and macrophages, keeping inflammation under control^11^. The related cytokine, TGF-β3, may also have similar anti-inflammatory properties^12^. TGF-βs are synthesized as a latent precursor molecules, which can be activated, for example, by proteolytic cleavage or by conformational changes caused by binding to thrombospondin-1 or to integrins. Integrin αvβ6 is a cell surface receptor for the TGF-β1 and -β3 latency-associated peptides, and a key activator of these cytokines in epithelial cells^13^.

Integrins are heterodimeric transmembrane cell adhesion and signaling receptors, which consist of one α and one β subunit^14^. In healthy adults, the expression of αvβ6 integrin is limited to some epithelial tissues, including hair follicles, intestinal epithelium and gingival JE^3^. However, its expression is highly induced in wound keratinocytes and in epithelial cancers^15–19^. Interestingly, inactivation of the *ITGB6* gene, which encodes the rate-limiting subunit of the αvβ6 integrin heterodimer formation, results in macrophage infiltration into the skin and accumulation of activated lymphocytes around conducting airways in the lungs in mice^20, 21^, suggesting that one of the functions of αvβ6 integrin is to constraint inflammation *in vivo*. A subsequent study showed that, specifically, it is the αvβ6 integrin-mediated localized activation of TGF-β1 that plays a major role in the anti-inflammatory surveillance *in vivo* ^11, 22^. Mice with a non-functional variant of the αvβ6 integrin-binding sequence in the TGF-β1 latency-associated peptide develop multi-organ mononuclear cell infiltrations^22^. Paradoxically, more generalized TGF-β1 activation (e.g. by proteolysis) may instead have pro-inflammatory effects in tissues, such as stimulating monocytes recruitment and activation^23, 24^.

In addition to αvβ6 integrin being an activator of TGF-β1, TGF-β1 activity is required for the maintenance of *ITGB6* expression in GECs^25^ with the *ITGB6* promoter having binding sites for Smad2/3 that serves as an intracellular transducer of TGF-β1 signaling^26^, indicating a mutual positive feedback loop between these two molecules. In healthy periodontal tissues, both αvβ6 integrin and TGF-β1 are constitutively expressed in the JE^25^, and it is likely that αvβ6 integrin-mediated activation of TGF-β1 is involved in the regulation of the anti-inflammatory response in these tissues. In periodontal disease, the expression of αvβ6 integrin becomes strongly downregulated in the PE, and patients with mutations in the β6 integrin gene can develop severe periodontal disease^25, 27^. In addition, mice deficient in β6 integrin spontaneously develop periodontal disease that involves PE formation, inflammation and bone loss^25^. Based on these observations, we have hypothesized that αvβ6 integrin-mediated TGF-β1 activation in JE is important for the maintenance of periodontal health^3^. However, the factors that decrease αvβ6 integrin expression in PE cells are not yet known.

In the present study, we investigated whether mature, multispecies oral bacterial biofilms are involved in αvβ6 integrin downregulation in GECs and whether αvβ6 integrin-deficiency affects their expression of inflammatory cytokines. We were able to show, for the first time, that biofilm components, including *Mycoplasma salivarium* lipopeptides, can indeed suppress β6 integrin mRNA and protein expression potentially by interfering with TGF-β1 signaling. In addition, we show that the reduced expression of this integrin promotes the induction of pro-inflammatory molecules by GECs.

## Results

### Integrin αvβ6 deficiency is associated with inflammation in periodontal disease

**Figure 1:**
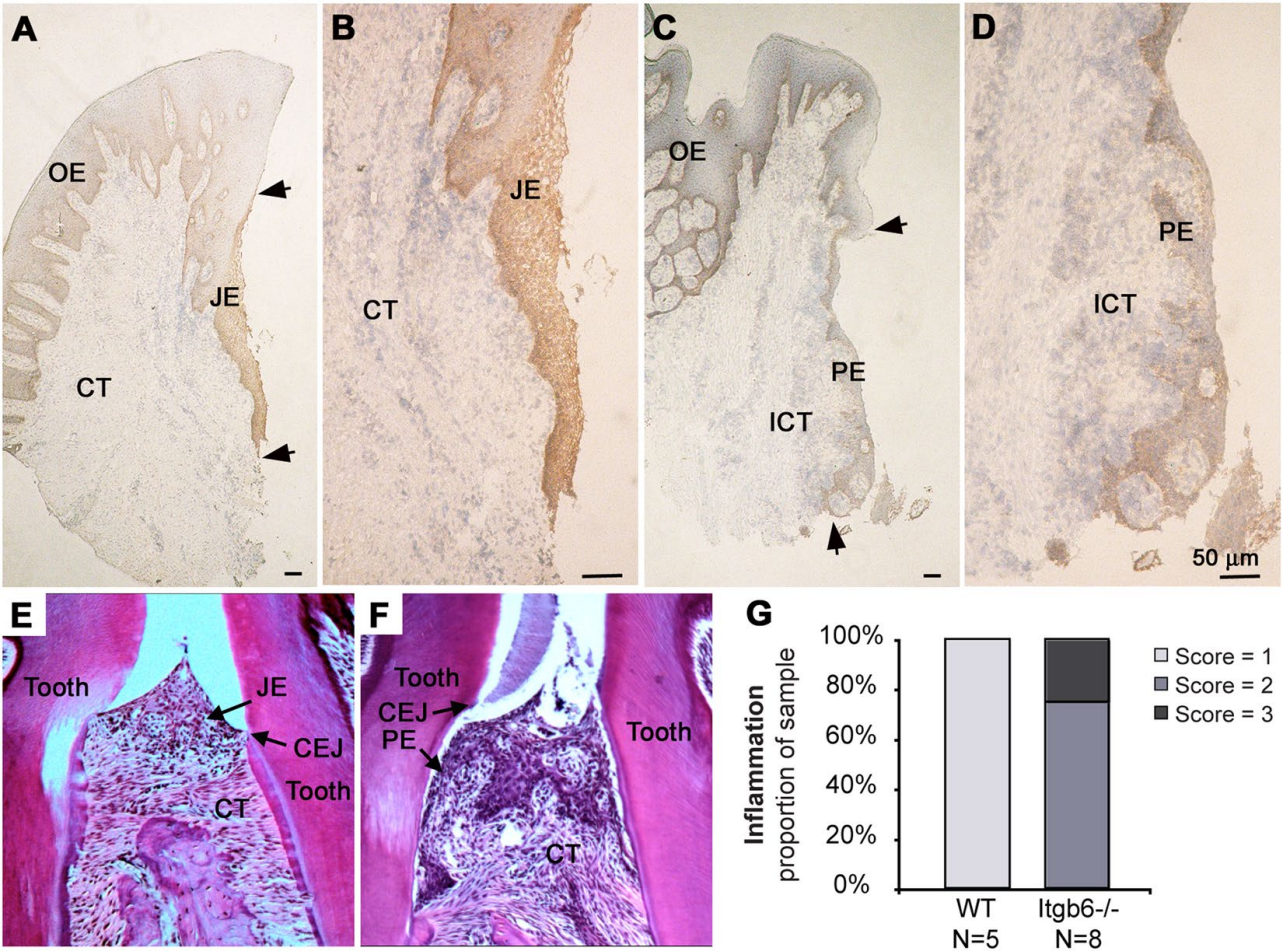
Expression of αvβ6 integrin in human and murine junctional epithelium (JE) and periodontal pocket epithelium (PE). (**A–D**) Expression αvβ6 integrin is expressed in healthy JE (**A** and **B**) but it becomes strongly downregulated in the PE in periodontal disease specimens (**C** and **D**). Arrowheads point the most coronal and apical part of the JE (**A**) and PE (**C**), respectively. (**E** and **F)** Hematoxylin-eosin-stained sections of periodontal tissues from WT and *Itgb6* ^−/−^ mice, respectively. (**G**) Periodontal inflammation scores of the WT and *Itgb6* ^−/−^ mouse gingiva. OE, Oral epithelium; ICT, Inflamed connective tissue; CEJ, Cemento-enamel junction.

We have previously shown that the expression of β1 and β4 integrins is variable in human periodontal disease, showing areas of focal loss and areas of increased expression^28^. In this earlier study, no expression of αv integrins was found in the PE. Consistent with these observations, our group later reported that the expression of αvβ6 integrin is strongly reduced in PE of human periodontal disease^25^. To assess whether this reduction in αvβ6 integrin levels is associated with periodontal inflammation, we further studied human JE/PE by immunohistochemistry. Integrin αvβ6 was strongly present in healthy JE (Fig. 1A,B) but showed diminished levels in PE of chronic periodontitis (Fig. 1C,D)^25^. A severe inflammatory reaction was observed adjacent to the αvβ6 integrin-depleted PE (Fig. 1D).

In WT mice, the JE between molar teeth was located against the enamel, ending at the cemento-enamel junction (CEJ) (Fig. 1E), whereas in *Itgb6* ^−/−^ mice, the JE had migrated below CEJ with numerous inflammatory cells present in the connective tissue underlying the JE (Fig. 1F). Quantification of the inflammatory response demonstrated that all *Itgb6* ^−/−^ specimens exhibited moderate to severe inflammation compared to minimal inflammation in WT animals (Fig. 1G). We then analyzed the expression of the other major epithelial integrins and *Il1b* in gingival tissue of WT and *Itgb6* ^−/−^ mice by RT-qPCR. Gene expression levels of αv, β1 and β4 (for basal epithelial integrin α6β4) integrins were not significantly different between WT and *Itgb6* ^−/−^ mouse gingiva (Supplementary Figure S1). However, *Il1b* expression was significantly higher in the gingiva of the *Itgb6* ^−/−^ mice compared to the WT animals (Supplementary Figure S1), confirming the increased inflammatory response in the αvβ6 integrin-deficient animals. The mRNA expression of other inflammatory cytokines tested was either low or showed no change between WT and *Itgb6* ^−/−^ (Supplementary Table S1). To further investigate whether the protein expression levels of β1 and α6β4 integrins showed any compensatory changes, we immunostained mouse gingival frozen sections with specific antibodies against these integrins. The localization and expression intensity of β1 and α6 integrins was similar in both *Itgb6* ^−/−^ and WT JE, whereas αvβ6 integrin was only detected in the WT mouse JE, as expected (Supplementary Figure S2).

Thus, the loss of αvβ6 integrin from JE is associated with periodontal inflammation, and reversely, its presence may offer protection against chronic inflammatory periodontitis. Furthermore, the loss of αvβ6 integrin does not appear to cause compensatory changes to other integrins.

### The expression of β6 integrin is reduced by heated dental plaque biofilm extract

**Figure 2:**
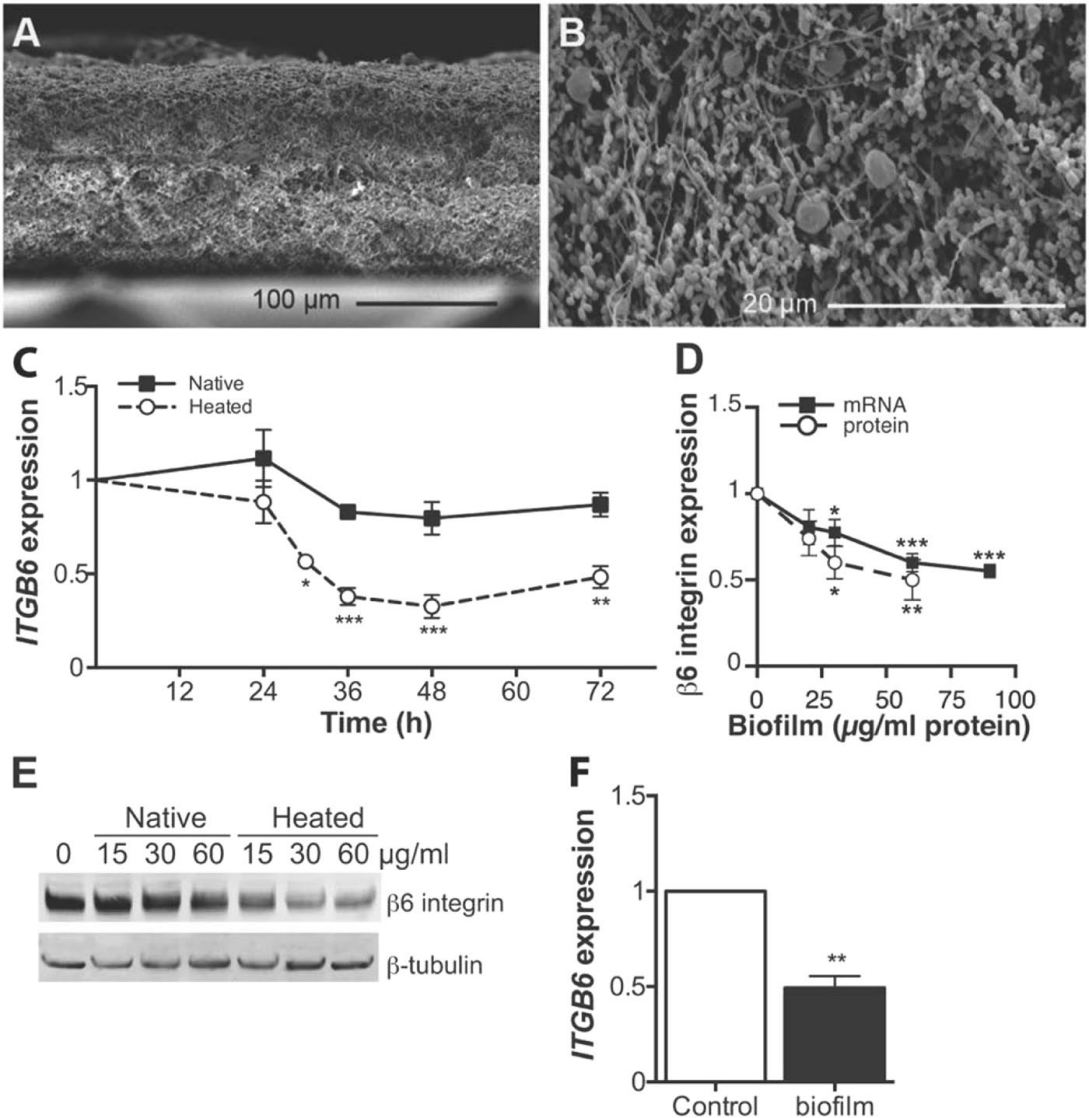
Structure of the bacterial biofilm and its effect on GEC β6 integrin expression. (**A** and **B**) Cross section SEM micrographs of multi-species oral bacterial biofilms cultured for three weeks showing the structural features of the biofilm. (**A**) scale bar = 100 μm; (**B**) scale bar = 20 μm. (**C**) The time course of β6 integrin mRNA expression in GECs treated with native or heated biofilm extract (#4 biofilm; 60 µg protein/ml) relative to untreated cells. Mean ± SEM of three experiments is presented. (**D**) Integrin β6 mRNA and protein expression in GECs treated with different amount of heated biofilm extract (#4 biofilm) for 32 and 48 h, respectively. The protein levels were quantified relative to β-tubulin. Mean ± SEM of five experiments is presented. (**E**) Representative Western blot image of the total β6 integrin protein levels in GECs treated with various doses of native or heated #4 biofilm extract for 48 h relative to β-tubulin. (**F**) Primary human GECs were exposed to heated oral biofilm extract (60 µg protein/ml) for 30 h and the relative gene *ITGB6* expression analyzed by RT-qPCR. Mean ± SEM of three experiments is presented. *p < 0.05; **p < 0.01; ***p < 0.001.

Because epithelial cells in periodontal pockets are exposed to bacterial biofilms, we hypothesized that biofilm components could be responsible for the decreased αvβ6 integrin levels in PE. To investigate this, a mature 3-week-old multispecies dental plaque biofilm with a thickness of about 150–200 µm was cultivated (Fig. 2A)^29^. Numerous bacterial filaments and coccoid bacteria were present together with spirilla and spirochetes (Fig. 2B), representing the typical, mature biofilm present in chronic periodontal disease^30^. SEM micrographs of multi-species oral bacterial biofilms from three additional donors showed similar thickness and complex bacterial ultrastructure (Supplementary Figure S3).

GEC exposure to live bacterial biofilms caused cytotoxicity (data not shown). Therefore, we treated the GECs with sonicated biofilm extracts, which did not cause overt changes to the cell appearance or growth during several days of culture (data not shown). Majority of the experimental data was obtained with biofilms from one donor, but key experiments were confirmed using biofilms from 3–4 different donors.

The GECs were first treated with biofilm extract (60 µg/ml of biofilm protein) for 0–72 h to determine the time course of *ITGB6* regulation. Since heating the biofilm extract may alter cellular response^25^, we tested both native and heat-treated extracts. We found that the heated extract suppressed *ITGB6* expression significantly after 30-h treatment in a concentration-dependent manner, whereas native extract did not (Fig. 2C,D). The total GEC β6 integrin protein level was similarly reduced after a 48-h treatment with heated biofilm, but not with native biofilm (Fig. 2D,E). The heated biofilm extract suppressed *ITGB6* expression in primary GECs similarly to the immortalized GECs (Fig. 2F), showing that this effect was not limited to the GEC cell line used in this study. Other major epithelial integrins were not significantly regulated by biofilm treatment; with biofilms from three different donors producing similar results (Supplementary Figure S4).

### Biofilm extract induces cytokine response in GECs

**Figure 3:**
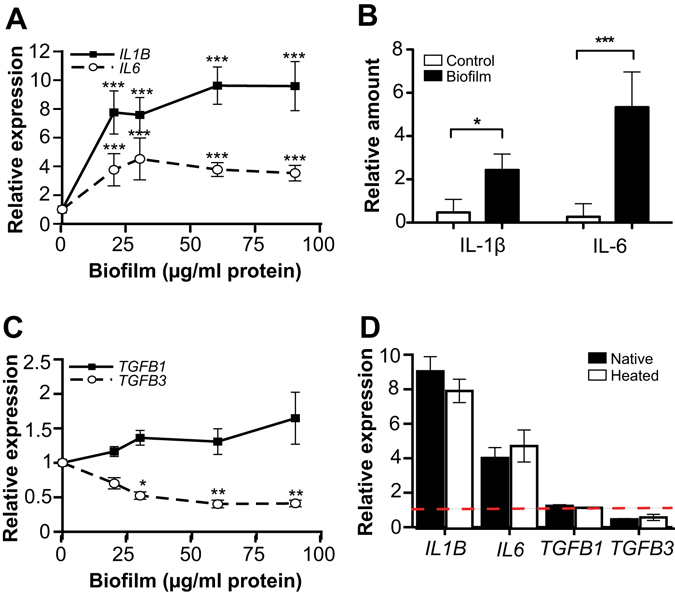
Inflammatory cytokine expression in biofilm-treated GECs. (**A** and **C)** GECs were exposed to various concentrations of oral biofilm extract (#4 biofilm; 0–90 µg protein/ml) for 32 h. RT-qPCR was then performed to assess the relative gene expression of (A) *IL1B* (n = 5) and *IL6* (n = 4) and (**C**) *TGFB1* (n = 6) and *TGFB3* (n = 4). Mean ± SEM is presented. (**B**) The amount of IL-1β and IL-6 secreted in the cell culture medium was determined by ELISA. (**D**) GECs were treated with either native or heated biofilm #4 extract (60 µg protein/ml) for 32 h and analyzed for *IL1B*, *IL6*, *TGFB1* and *TGFB3* expression by RT-qPCR (n = 3–5). Dashed line indicates control expression level. *p < 0.05; **p < 0.01; ***p < 0.001.

Next, we explored the potency of the biofilm extracts to regulate the gene expression of pro- and anti-inflammatory cytokines in GECs. The mRNA expression and protein secretion of pro-inflammatory IL-1β and IL-6 were significantly increased by biofilm treatment (Fig. 3A,B). The mRNA expression of the anti-inflammatory TGF-β1 was modestly upregulated but without reaching statistical significance, whereas TGF-β3 gene expression was significantly reduced (Fig. 3C). Comparable results obtained with biofilms from four different donors are shown in Supplementary Figure S5. GECs cytokine response to both native and heated biofilm extracts was similar (Fig. 3D). The mRNA expression of other inflammatory cytokines expressed by epithelial cells was either low or showed no change after biofilm treatment (Supplementary Table S2). Thus, the biofilm extract strongly induced pro-inflammatory cytokine expression in the GECs in a dose-dependent manner, whereas its effect on anti-inflammatory cytokine expression was modest or inhibitory. Therefore, the increasing amounts of biofilm may shift the balance towards the dominance of pro-inflammatory cytokine production in GECs.

### Integrin αvβ6-deficient keratinocytes are hyper-responsive to biofilm-induced pro-inflammatory cytokine regulation

**Figure 4:**
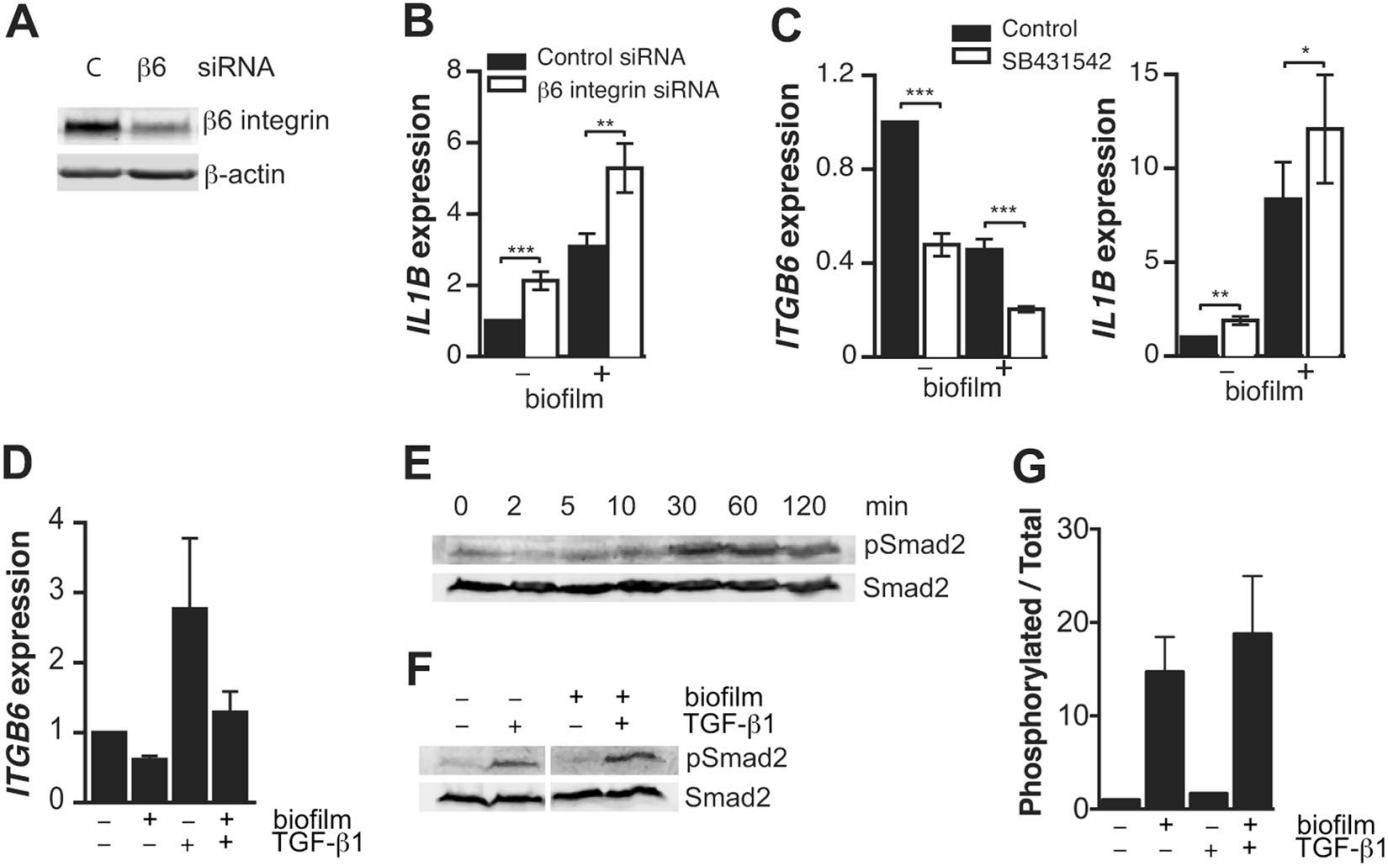
The effect of β6 integrin-deficiency on inflammatory cytokine expression. (**A**) SiRNA knockdown of β6 integrin in the GECs after a 48-h transfection relative to β-actin detected by Western blotting. SiRNA that is not homologous to any human gene (**C**) was used as a control. (**B**) The effect of β6 integrin knockdown on *IL1B* expression in biofilm-treated (60 µg protein/ml; 32 h) and non-treated GECs determined by RT-qPCR. Mean ± SEM is presented, n = 3. (**C**) The effect of TGF-β1 signaling inhibitor SB431542 (5 µM) on *ITGB6* and *IL1B* expression in biofilm-treated (60 µg protein/ml; 32 h) and non-treated GECs. Mean ± SEM, n = 3. (**D**) GECs were treated with TGF-β1 (2 ng/ml), heated biofilm extract (60 µg protein/ml) or a combination of both for 32 h and analyzed for *ITGB6* expression by RT-qPCR. Mean ± SEM, n = 3. (**E**) GECs were treated with TGF-β1 (2 ng/ml) for 0–120 min and analyzed for Smad2 phosphorylation (activation) relative to total Smad2 by Westerm blotting. (**F** and **G**) GECs were treated with TGF-β1 (2 ng/ml), heated biofilm extract (60 µg protein/ml) or a combination of both for 30 min and analyzed for Smad2 phosphorylation relative to total Smad2 by Westerm blotting. The ratio of phosphorylated Smad2 to total Smad2 was determined from triplicated experiments. *p < 0.05; **p < 0.01; ***p < 0.001.

We determined whether the αvβ6 integrin-deficiency itself could cause an altered cytokine response in GECs by treating the cells with *ITGB6* siRNA, which resulted in over 90% reduction in β6 integrin protein levels (Fig. 4A). *ITGB6*-knockdown-cells expressed significantly (2-fold) more IL-1β mRNA than negative control-transfected cells both with or without biofilm extract treatment (Fig. 4B). These results indicate that the GECs with reduced β6 integrin expression show an enhanced inflammatory response.

### Inhibition of TGF-β1 signaling suppresses β6 integrin expression and increases IL-1β expression

Since epithelial αvβ6 integrin and TGF-β1 are coupled in a positive feedback loop where αvβ6 integrin activates TGF-β1 and TGF-β1 signaling in turn maintains αvβ6 integrin expression^13, 25^, we tested the effect of TGF-β receptor inhibitor SB431542 on GEC *ITGB6* and *IL1B* expression. The inhibitor treatment significantly reduced *ITGB6* expression and increased *IL1B* expression both in control and biofilm-treated cells (Fig. 4C). Furthermore, heated biofilm could attenuate TGF-β1-induced *ITGB6* expression in GECs (Fig. 4D). Thus, dental plaque bacteria may suppress β6 integrin by interfering with GEC TGF-β1 signaling. We then tested whether the biofilm treatment interfered with TGF-β1-induced Smad2 activation in GECs. Maximal activation based on increased Smad2 phosphorylation was achieved after 30-min treatment with TGF-β1 (Fig. 4E). Biofilm did not directly affect Smad2 activation (Fig. 4F,G), indicating a more downstream inhibition of this signaling pathway.

### Diacylated lipopeptide FSL-1 downregulates β6 integrin expression in GECs

Cells recognize prokaryotic pathogens via pattern-recognition receptors of the innate immune system, such as toll-like receptors (TLRs) and nucleotide-binding oligomerization domain (NOD)-like receptors^31, 32^. TLRs 1-9 as well as NOD1 and NOD2 are expressed in human gingival epithelium^33, 34^. Additionally, TLR2 expression has been detected in PE^35^. To determine whether these receptors were involved in biofilm-induced *ITGB6* suppression, we analyzed TLR1–10 and NOD1 and -2 expression in GECs by RT-qPCR and detected mRNA for TLRs 2–6 and NOD1 (Table 1). As TLRs 2, 4 and 5 are major receptors for bacterial components, we further explored their presence on the GEC cell surface by flow cytometry. GECs expressed TLR2 and TLR5, whereas TLR4 was not detected (Table 1).

**Table 1:** Toll-like receptors in gingival epithelial cells.

Receptor	Ligand^31, 32^	RT-qPCR	Flow cytometry	Ligands
TLR1	TLR2/1 heterodimer: native mycobacterial lipoprotein, triacylated bacterial lipopeptides	Inconclusive		Pam3CSK4 (300 ng/ml)
TLR2	Gram(+) peptidoglycan, lipoarabinomanan, lipoproteins, lipoteichoic acid (LTA), lipopolysaccharide (LPS) of some gram(−) bacteria, spirochete and fungi	Expressed	Expressed	LTA (2 µg/ml)
TLR3	Double-stranded viral RNA	Expressed		
TLR4	Gram(−) LPS	Expressed	Not detected	
TLR5	Bacterial flagellin	Expressed	Expressed	Flagellin (100 ng/ml)
TLR6	TLR2/6 heterodimer: diacylated mycoplasmal lipopeptides	Expressed		FSL-1 (100 ng/ml) MALP-2 (100 ng/ml)
TLR7	Single-stranded viral RNA	Inconclusive		
TLR8	Single-stranded viral RNA	Not detected		
TLR9	CpG motifs in bacterial DNA	Not detected		
TLR10	TLR2/10 heterodimer: Listeria; a modulatory receptor with mainly inhibitory properties against other TLR2 ligands	Not detected		
NOD1	Bacterial peptidoglycans	Expressed		
NOD2	Bacterial peptidoglycans	Not detected		

**Figure 5:**
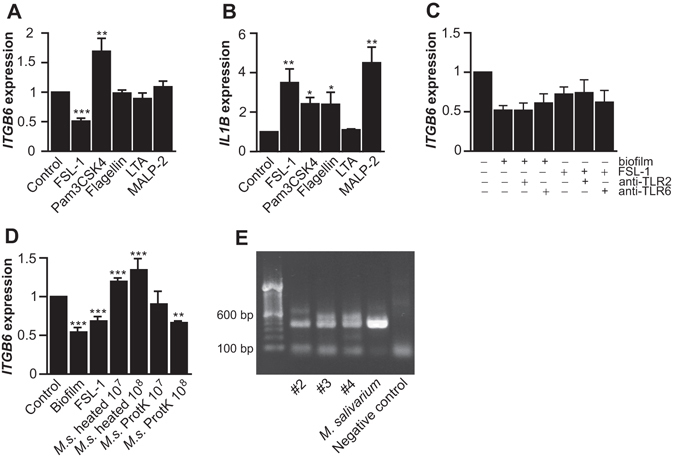
The role of TLR ligands in the downregulation of β6 integrin in GECs. (**A**) and (**B**) GECs were treated with FSL-1 (100 ng/ml; n = 4), Pam3CSK4 (300 ng/ml; n = 4), flagellin (100 ng/ml; n = 3), LTA (2 µg/ml; n = 3) or MALP-2 (100 ng/ml; n = 3) or left untreated for 32 h, and their effect on *ITGB6* (**A**) and *IL1B* expression (**B**) was analyzed by RT-qPCR. Mean ± SEM is presented. (**C**), GECs were pre-treated with anti-TLR2 and anti-TLR6 blocking antibodies for 1 h, after which heated biofilm extract (60 µg protein/ml) or FSL-1 (100 ng/ml) was added for 32 h. *ITGB6* expression was analyzed by RT-qPCR. Mean ± SEM is presented (n = 3–5). (**D**) GECs were treated with heated *Mycoplasma salivarium* extract (10^7^ or 10^8^ cfu/ml) with or without proteinase K digestion for 32 h and their *ITGB6* expression was analyzed by RT-qPCR. Heated biofilm extract (60 µg protein/ml) and FSL-1 (100 ng/ml) were used as positive controls. Mean ± SEM of three experiments is presented. *p < 0.05; **p < 0.01; ***p < 0.001. (**E**) Mycoplasma was detected in oral bacterial biofilms (#2, #3 and #4 shown) by PCR and agarose gel electrophoresis. *M. salivarium* and water were used as a positive and negative control, respectively.

To specifically determine whether biofilm-induced αvβ6 integrin downregulation is mediated by TLR2 or TLR5 NOD1, we treated GECs with small molecule ligands for these receptors and assessed *ITGB6* expression. To this end, we treated GECs with Pam3CSK4 (triacylated lipopeptide; 300 ng/ml; ligand for TLR1/2), FSL-1 (diacylated lipopeptide; 100 ng/ml; ligand for TLR2/6) and flagellin (100 ng/ml; ligand for TLR5). We also tested lipoteichoic acid (LTA; 2 µg/ml), a TLR2/lectin pathway ligand (Table 1)^36^. Interestingly, Only FSL-1 was able to reduce *ITGB6* significantly. In contrast, Pam3CSK4 upregulated *ITGB6* expression while flagellin had no effect (Fig. 5A). Based on these results, we tested another diacylated lipopeptide, MALP-2. Interestingly, unlike FSL-1, this molecule had no effect on *ITGB6* expression. In contrast, Pam3CSK4, FSL-1, flagellin and MALP-2 all induced IL-1β expression, indicating that the GECs were able to respond to these ligands (Fig. 5B). LTA had no effect on the expression of either *ITGB6* or *ILB1* in GECs (Fig. 5A,B), but induced IL-1β and IL-6 gene expression in mouse macrophages (Supplementary Figure S6), confirming the biological activity of the molecule. ML130, a chemical NOD1 inhibitor, was not able to block the biofilm-induced *ITGB6* suppression in GECs, indicating that NOD1 does not play a major role in this process (Supplementary Figure S6). To further test whether the biofilm-induced downregulation of *ITGB6* could involve TLR2/6, we used specific function blocking antibodies against this receptor. Surprisingly, neither TLR2 nor TLR6 blocking antibodies (up to 20 µg/ml) inhibited the biofilm- and FSL-1-mediated *ITGB6* suppression (Fig. 5C), indicating that this effect may be mediated via another pathway.

### *Mycoplasma* species are components of *in vitro* dental plaque biofilms

Since FSL-1 represents the N-terminal lipopeptide of cell membrane lipoprotein LP44 found on the cell membrane of a common oral bacterium, *Mycoplasma salivarium* ^37^, we tested whether *M. salivarium* might be among the sub-gingival plaque microbes capable of downregulating *ITGB6*. Only when *M. salivarium* extract was first treated with proteinase K, which releases the lipopeptide from the bacterial cell surface^37^, *ITGB6* was downregulated similarly to biofilm extract and FSL-1 (Fig. 5D), indicating that *ITGB6* suppression could only be obtained by the soluble ligand. As a comparision, we tested whether periodontal pathogens *Porphyromonas gingivalis* and *Treponema denticola* could suppress *ITGB6* expression on GECs. The native, heated or proteinase K-treated extracts of these bacteria all failed to downregulate *ITGB6* expression in GECs while still inducing *IL1B* expression (Supplementary Figure S7).

To further explore whether *M. salivarium* could participate in the biofilm-induced *ITGB6* suppression, we analyzed bacterial biofilms from three different donors for the presence of *Mycoplasma* by PCR. All three biofilms were positive for *Mycoplasma* species and showed a band size representative for *M. salivarium* (Fig. 5E). The total genomic copies of oral *Mycoplasma* 1S rRNA gene were about 1–2% of total genomic copies of the universal bacterial gene in oral bacteria biofilms (Supplementary Figure S8). The actual relative proportion of *Mycoplasma* of the total number of bacteria in the biofilms will depend on the species composition of the given biofilm, as the copy number of rRNA operons per bacterial genome varies between different bacterial species^38^.

## Discussion

The presence of αvβ6 integrin in JE seems essential for periodontal health through its maintenance of the anti-inflammatory TGF-β1 signaling, and, reciprocally, TGF-β1 activity sustains αvβ6 integrin expression in GECs^25, 28^. Accordingly, *ITGB6* expression could be suppressed in GECs by blocking TGF-β1 signaling with an inhibitor of TGF-β receptor kinase activity (SB431542). Also excess soluble TGF-β1-LAP or a function-blocking anti-TGF-β1 antibody suppress *ITGB6* expression^25^. However, the cause of αvβ6 integrin loss in periodontal disease is unclear. In the present study, we explored whether oral bacterial biofilms may be responsible for its suppression in GECs. Our results show that prolonged exposure to heated extracts of oral multispecies bacterial biofilms and to a soluble diacylated lipopeptide from *M. salivarium* significantly downregulated the expression of β6 integrin mRNA and protein in GECs, potentially by interfering with their TGF-β1 signaling. Furthermore, this β6 integrin deficiency was associated with increased pro-inflammatory response, manifested by induction of pro-inflammatory cytokines IL-1β and IL-6. Other bacterial cell surface components and extracts of classic periodontal pathogens *P. gingivalis* and *T. denticola* failed to suppress *ITGB6* expression. We have also shown previously that cytokines associated with periodontal disease or purified lipopolysaccharides from *P. gingivalis*, *Tannerella forsythia* and *T. denticola* were unable to suppress *ITGB6* expression^25^. In a recent *in vitro* wound healing study, *ITGB6* expression was, however, reduced in oral keratinocytes exposed to live, planktonic *P. gingivalis* and *Fusobacterium nucleatum* ^38, 39^. Also, we have shown previously that native *P. gingivalis* cell extract can reduce the TGF-β1-induced increase in theαvβ6 integrin cell surface levels, likely via the proteolytic action of bacterial gingipains, as this effect was abolished after heating of the extract^25^. In the present study, however, only the heated biofilm extract suppressed the β6 integrin mRNA and protein levels in GECs, indicating a different mechanism. It is, therefore, possible that the different bacterial species present in multispecies bacterial biofilms in periodontal pockets can synergistically contribute the loss of αvβ6 integrin via several means simultaneously.

*Mycoplasma* species lack the classical immunomodulatory cell wall components, such as lipopolysaccharides, LTA and peptidoglycans, with lipoproteins or -peptides being their most important pathogenic elements^40^. Oral *Mycoplasma* species have been detected in over 90% of saliva samples from subjects with or without oral diseases and are traditionally regarded as non-pathogenic – *M. salivarium* being the most common species^41, 42^. However, its significantly higher prevalence in the subgingival biofilms in the periodontally-diseased compared to healthy subjects correlates with the severity of periodontal disease, suggesting its involvement in the disease process^43–46^. Oral plaque bacteria function as an integrated community where properties of the same organisms may differ from their behavior in planktonic culture^47^. Therefore, interplay of different bacteria in the biofilm may determine its pathogenicity. Our biofilms were derived from healthy donors, but prolonged culturing in anaerobic conditions may lead to selective growth of harmful bacteria from the normal oral microflora, making the resulting biofilms reminiscent of the biofilms in periodontal pockets.

High protease activity is a major characteristic of the classical periodontal pathogens, such as *P. gingivalis* (gingipains), *T. forsythia* (PrtH) and *T. denticola* (dentilisin), causing damage to epithelial barrier and allowing harmful, immunostimulatory bacteria or their components to translocate deeper into the gingival tissue to elicit inflammatory response and bone loss^48^. In this study, only *M. salivarium* extract digested with proteinase K was able to suppress *ITGB6* expression. Proteinase K digestion releases the N-terminal lipopeptide, similar to FSL-1, of *M. salivarium* lipoprotein LP44^37^. The host cells can recognize minute quantities of soluble lipoproteins, whereas in intact bacteria, the active portion of the molecule may be inaccessible to the host receptors^49^. It is possible that the periodontal pathogen or host proteases in the gingival tissue may release the bioactive *M. salivarium* lipopeptides to suppress *ITGB6* expression. Intracellular *M. salivarium* has been recently detected in human oral leukoplakia lesions^50^. It remains to be documented whether these bacteria could also be present within PE in human periodontitis.

It is generally thought that diacylated lipopeptides signal via TLR2/6 heterodimers, with both the lipid and peptide parts contributing to ligand specificity^51, 52^. The framework structure of the diacylated mycoplasma lipopeptides FSL-1 and MALP-2 are the same but they differ in their amino acid sequence and length of the peptide portion^53^. Only FSL-1 was able to downregulate *ITGB6* expression, suggesting that the peptide portion of the molecule mediated the effect. Interestingly, function-blocking antibodies against either TLR2 or TLR6 failed to prevent the biofilm- or FSL-1-induced *ITGB6* suppression, indicating that this effect may be TLR2/6 independent in GECs.

Only the expression of β6 integrin subunit was downregulated by the bacterial biofilm extract while the expression of other major epithelial integrin subunits was not significantly changed, suggesting that this change was specific for β6 integrin. Accordingly, our previous immunohistochemical studies have indicated that the expression of β1 and α6β4 integrins in human PE is variable with areas of reduced and increased expression^28^. Their expression in *Itgb6* ^−/−^ mice also remanined unchanged, indicating that they are not critically involved in the progression of periodontal disease.

In summary, in the present study, we demonstrate, for the first time, that components of multispecies bacterial biofilms can downregulate αvβ6 integrin levels, likely by suppressing TGF-β1 signaling, leading to increased production of pro-inflammatory cytokines known to participate in the initiation and progression of periodontal disease. Therefore, we propose a new paradigm, in which epithelial cells, through their αvβ6 integrin-mediated TGF-β1 activation, play a crucial protective role in JE against periodontal disease. Understanding the mechanisms of αvβ6 integrin suppression during the disease progression may provide strategies and therapeutic targets to prevent the loss of this receptor and, consequently, to treat periodontal disease.

## Methods

### Ethics approval for human and animal studies

The research involving human subjects was conducted in accordance with Canada’s national guidelines, the Tri-Council Policy Statement on Ethical Research Involving Humans (TCPS2) and adhered to the principles of WMA Declaration of Helsinki - Ethical Principles for Medical Research Involving Human Subjects. The Clinical Research Ethics Board at the University of British Columbia reviewed and approved all procedures involving human tissue donors (Protocol Number C03-0297). Gingival tissue samples were collected as a part of routine dental care. Informed written consent was obtained from the patients providing the tissue samples according to the Helsinki Declaration (1975).

The research involving animals adhered to the guidelines provided by Canadian Council on Animal Care. The University of British Columbia Committee on Animal Care approved all animal procedures used in this study (Protocol Numbers A05-0142, A13-0316 and A16-0034).

### Materials

Bacterial ligands used were FSL-1 (Pam2CGDPKHPKSF; a synthetic diacylated lipopeptide derived from *M. salivarium;* Abcam, Cambridge, MA, USA), Pam3CSK4 (Pam3CSKKKK; a synthetic triacylated lipopeptide; Tocris Bioscience; Bristol, UK), flagellin (full-length from *Salmonella typhimurium* produced in *Escherichia coli*; Abcam), LTA-BS (lipoteichoic acid from *Bacillus subtilis*; Sigma-Aldrich Canada, Oakville, ON, Canada), MALP-2 (Pam2CGNNDESNISFKEK; a synthetic diacylated lipopeptide derived from *M. fermentans*; Novus Biologicals, Oakville, ON, Canada). NOD1 inhibitor ML130 was from Selleckchem Chemicals (Houston, TX, USA). TGF-β1 and TGF-β1 signaling inhibitor SB431542 were from EMD Millipore (Etobicoke, ON, Canada) and Selleckchem Chemicals, respectively. Function-blocking antibodies against human TLRs were: TLR2 (PAb hTLR2; InvivoGen and TL2.1; eBioscience Inc.; San Diego, CA, USA) and TLR6 (PAb hTLR6; InvivoGen). Primary antibodies used for Western blotting were: β6 integrin (AF2389; R&D Systems, Inc., Minneapolis, MN, USA), β-actin (Ab8227; Abcam), β-tubulin (MAB3408; EMD Millipore), phospho-Smad2 (#3101) and Smad2 (#3103; both from Cell Signaling Technology, Denvers, MA, USA). Primary antibodies used for flow cytometry were: TLR4 (HTA125; eBioscience), TLR2 (TL2.1) and TLR5 (Q2G4; InvivoGen). All PCR primers were synthesized by Integrated DNA Technologies, Inc. (Coralville, IA, USA).

### Human tissue specimens and immunohistochemistry

Gingival tissue samples with normal gingival apparatus (healthy control) and from periodontal disease patients (deep pockets exceeding 5 mm) were collected from routine dental care, placed in Tissue-Tek^®^ (OCT compound, Sakura Finetek USA, Inc., Torrance, CA, USA) and snap-frozen in liquid nitrogen. Frozen sections (6–8 μm) were cut with a cryostat and stored at −80 °C until used for immunolocalization studies. To this end, frozen sections were fixed with acetone (−20 °C) for 5 min, rinsed and incubated in normal blocking serum (Vectastain Elite ABC Kit; Vector Laboratories, Inc., Burlingame, CA, USA) in a humidified chamber at room temperature for 30 min. After rinsing, the sections were incubated overnight with the primary antibody against αvβ6 integrin (β6B1; a generous gift from Dr. Dean Sheppard, University of California, San Francisco, CA, USA), followed by an incubation with a biotinylated, horseradish peroxidase-conjugated secondary antibody (Vectastain) for 1 h, incubation with ABC avidin/peroxidase reagent and reaction with Vector DAB Peroxidase Substrate Kit (Vector Laboratories). To stop the reaction, the sections were rinsed with distilled water for 10 min. After counterstaining with hematoxylin, the sections were allowed to air dry, mounted using Vectamount permanent mounting medium (Vector Laboratories), viewed and photographed using a light microscope.

### Histological assessment of mouse jaw samples

Maxillae were removed from sacrificed wild-type (WT) and *Itgb6* ^−/−^ mice (both FVB/N strains; *Itgb6* ^−/−^ a kind gift from Dr. Dean Sheppard, University of California, San Francisco, CA, USA) and immediately fixed in 4% formaldehyde in phosphate-buffered saline (PBS, pH 7.4) for two days and then decalcified in PBS containing 2% formaldehyde and 0.4 M EDTA for five weeks. The decalcified samples were dehydrated, embedded in paraffin, cut in sections (8 μm) and stained with hematoxylin and eosin. Level of interproximal inflammation between the first and second maxillary molars was assessed from the digital images using a visual scoring system (1, mild; 2, moderate; 3, severe).

### Preparation of multispecies oral *in vitro* biofilms

Oral bacterial biofilms were cultured as previous described^29, 54^. Sterile hydroxyapatite discs (9.7-mm diameter by 1.5-mm thickness; Clarkson Chromatography Products, Williamsport, PA, USA) were used as the biofilm growth substrate. The discs were coated with bovine dermal type I collagen (10 µg/ml collagen in 0.012 N HCl; Cohesion, Palo Alto, CA, USA) for overnight. The collagen-coated discs were placed in 24-well tissue culture plate wells containing 1.8 ml of brain heart infusion broth (BHI; Difco, Detroit, MI, USA). Subgingival plaque on the first or second upper molars was collected from four healthy volunteers and mixed in BHI separately. Each well was inoculated with 0.2 ml of dispersed dental plaque, containing a minimum bacterial cell count of 3.2 × 10^7^ colony-forming units/ml. The bacteria were incubated under anaerobic conditions (AnaeroGen; Oxoid, Cambridge, UK) at 37 °C for 21 days, changing the growth medium once a week. The biofilms from each donor was cultured 1–5 times, each time from a fresh plaque sample. Most experiments were performed with biofilm from one donor (#4), but key experiments were confirmed using biofilms from 3–4 different donors.

After 21 days of culture, two discs were cut into four sections and measured for biofilm thickness by scanning electron microscopy (SEM; Stereoscan 260; Cambridge Instruments, Cambridge, UK). Samples were pre-fixed with phosphate-buffered 2.5% glutaraldehyde for 30 minutes before further fixation in 1% OsO_4_ for 1 h. The specimens were dehydrated in increasing concentrations of ethanol (50%, 70%, 80% and 100%) and then dried using a critical point drier (Samdri-795; Tousimis Research Corporation, Rockville, MD, USA), sputter-coated with gold palladium (Hummer VI; Technic Inc, Anaheim, CA, USA), and examined by SEM. The thickness of three random areas of the biofilm on each piece was measured by using ImageJ software (ImageJ 1.34n; National Institutes of Health, Bethesda, MD, USA).

To prepare the biofilm extracts, the discs were first gently dipped in PBS to remove the bacterial culture medium without disturbing the biofilm, after which the bacteria were dispersed into PBS by vigorous pipetting, snap-frozen and ground over liquid nitrogen using a mortar and pestle. The bacterial powder was then collected and sonicated five times with Branson Sonifier 250 (Branson Ultrasonics Corp., Danbury, CT, USA) on ice (5-second sonication at a power output of 3 and duty cycle of 10%), then centrifuged by 12,000 g for 10 min to remove the insoluble matter at +4 °C. The supernatant was aliquoted and stored at −20 °C. The Bio-Rad Protein Assay reagent (Bio-Rad Laboratories, Hercules, CA, USA) was used for quantification of the total protein concentration of each biofilm extract. The biofilm thickness, structure and viability were measured using scanning electron microscopy (SEM) and confocal microscopy^29^.

*M. salivarium* (strain H110; ATCC 23064-TTR) was purchased from Cedarlane (Burlington, ON, Canada). The bacteria were collected by centrifugation (12,000 g for 30 min at +4 °C), washed once with PBS, re-centrifuged and suspended in PBS (10^9^ cells/ml). Bacterial extracts were prepared as above for the biofilms. To release the cell membrane lipopeptide similar to FSL-1 (see above), some of the *M. salivarium* extract was treated with 1 µg/ml proteinase K (Promega, Madison, WI, USA) at +37 °C for 2 h, as previously described^37^, followed by boiling for 5 min to inactivate the enzyme.

### Exposure of epithelial cells to bacterial biofilms, bacterial components and TGF-β1

Spontaneously immortalized human gingival epithelial cells (GECs)^55^ were maintained in Dulbecco’s modified Eagle’s medium (DMEM, Gibco, Life Technologies, Inc., Burlington, ON, Canada) supplemented with 23 mM sodium bicarbonate, 20 mM HEPES, 1% antibiotics (50 µg/ml streptomycin sulfate, 100 U/ml penicillin; Gibco) and 10% heat-inactivated fetal bovine serum (FBS; Gibco). Primary human GECs were grown from explants of gingival biopsies obtained from a healthy patient with no gingival inflammation as previously described^56^, and the contaminating fibroblasts were removed from the explant cultures by mild trypsinization. The primary GECs were maintained in EpiLife medium (Gibco) supplemented HKGS supplement, 1% antibiotics and 10 µM Y-27632 (Selleckchem) to maintain a stem cell-like non-differentiated phenotype^57^. The cell cultures were routinely confirmed to remain mycoplasma-free by PCR (PCR Mycoplasma Detection Kit; Applied Biological Materials, Inc., Richmond, BC, Canada). The same test was used for determining the presence of mycoplasma in the bacterial biofilms.

GECs were seeded into plates at 5 × 10^4^ cells/cm^2^ for 48 h in their complete growth medium, rinsed once with PBS, switched to FBS-free medium and treated with different concentrations of biofilm extract (heat-treated for 5 min at 95 °C or not heated) or bacterial components for 0–72 h. The cells were then rinsed with PBS and used for RNA isolation and quantitative real-time reverse transcription PCR (RT-qPCR) or for Western blotting. Medium cytokine levels were analyzed by ELISA (Multi-Analyte ELISArray^TM^ kit; SABiosciences Corp, Frederick, MD, USA). Cell surface receptor expression was analyzed by flow cytometry as previously described^58^. To explore the involvement of TGF-β1 signaling in *ITGB6* suppression, GECs were pre-treated with 5 µM SB431542 or left untreated for 1 h before adding the biofilm extract. To study the effect of biofilm on GEC TGF-β1 signaling, the cells were left untreated or treated with TGF-β1 (2 ng/ml) and/or heated biofilm extract (60 µg/ml of biofilm protein) and analyzed for Smad2 activation (phosphorylation) by Western blotting or for *ITGB6* expression by RT-qPCR.

### RT-qPCR

Total RNA was extracted from the treated gingival epithelial cells (GECs) using NucleoSpin RNA II kit (Macherey-Nagel, Inc., Bethlehem, PA, USA), and the total RNA concentration was measured by spectrophotometry at 260 nm. Total RNA (1 µg) was reverse-transcribed with iScript Select cDNA synthesis kit (Bio-Rad) according to the manufacturer’s instructions. Each RT product was diluted to a concentration with a threshold-cycle value well within the range of its standard curve. Amplification reactions were conducted in triplicates for target genes using asparagine-linked glycosylation 9 (*ALG9*), beta-2-microglobulin (*B2M*) and glyceraldehyde-3-phosphate dehydrogenase (*GAPDH*) as reference genes. PCR primer sequences are listed in Supplementary Table S3. 5 µl of each diluted RT product was mixed with 10 µl of 2 × iQ SYBR Green I Supermix (Bio-Rad) and 5 pmol of primers in a final volume of 20 µl. Real-time PCR amplification was performed with the CFX96 system (Bio-Rad; program: 3 min at 95 °C, followed by 45 cycles of 15 s at 94 °C, 20 s at 60 °C and 20 s at 72 °C). The data were analyzed with Gene Expression Analysis for CFX Manager Software Version 2.1 (Bio-Rad). PCR detection level was adjusted to ≥1.5-fold to reflect a significant change. Targets with Cq values indistinguishable from no-cDNA negative controls were considered ‘not detected’.

### Western blotting

The cells were washed once with PBS and lysed in 1x Laemmli sample buffer. Cell lysates were separated by SDS/PAGE and transferred onto Amersham Protran nitrocellulose membrane (GE Healthcare, Little Chalfont, Buckinghamshire, UK). The membranes were blocked in Odyssey Blocking Buffer (LI-COR Biosciences, Lincoln, NE, USA) for 1 h and then incubated with primary antibodies (see above) at +4 °C for overnight. After incubation with species-appropriate IRdye-conjugated secondary antibodies (LI-COR), the blots were scanned with LI-COR Odyssey Infrared Imaging system, and the results analyzed with Odyssey software version 3.0.

### RNA interference

In a set of experiments, β6 integrin was knocked down in GECs by RNA interference prior to treatment with the biofilm extract. The small interfering RNA (siRNA) against *ITGB6* (UGGGCUGACAAGUAAUUCCdTdT)^59^ and negative control siRNA (ACUUCGACACAUCGACUGCdTdT; not homologous to any human gene) were synthesized by Invitrogen (Carlsbad, CA, USA). For each siRNA transfection, 62.5 pmol of siRNA oligonucleotide dimers (final concentration 25 nM) and 3.8 µl of Lipofectamine RNAiMAX (Invitrogen) were diluted separately in 250 µl of DMEM, combined and added to GEC cell suspension (5 × 10^5^ in 2 ml of FBS-free, antibiotic-free (DMEM). The cells were gently shaken with the liposome-siRNA complexes at room temperature for 30 min and then plated in 6-well plate wells. After three-hour incubation at 37 °C (5% CO_2_) to allow cell adhesion, FBS was added to the cells to final concentration of 8%. After 48 h, the GECs were washed once with PBS, switched to FBS-free DMEM, treated with 60 µg/ml of biofilm extract protein for 32 h, 48 h or left untreated, and then harvested for RT-qPCR or Western blotting, respectively.

### Statistical analysis

Experiments were repeated separately at least three times. Student’s t-test for paired comparisons or one-way ANOVA followed by Tukey’s post hoc test for multiple comparisons was performed. Statistical analysis for RT-qPCR data (preformed in triplicates) was done using log2-transformed data^60^. Statistical significance was set at p < 0.05 and also required a minimum of 1.5-fold change.

## Electronic supplementary material

Supplementary material
