# Supplementary material for: Suppression of αvβ6 Integrin Expression by Polymicrobial Oral Biofilms in Gingival Epithelial Cells
Source: Sci Rep. 2017 Jun 30;7:4411. doi: 10.1038/s41598-017-03619-7 (PMC5493688; doi:10.1038/s41598-017-03619-7)

# Suppression of αvβ6 Integrin Expression by Polymicrobial Oral Biofilms in Gingival Epithelial Cells

**Jiarui Bi, Leeni Koivisto, Aihui Pang, Ming Li, Guoqiao Jiang, Saljae Aurora, Zhejun Wang, Gethin R. Owen, Jiayin Dai, Ya Shen, Daniel Grenier, Markus Haapasalo, Lari Häkkinen, Hannu Larjava**

## Supplementary information

## Methods

### RT-qPCR on mouse gingiva

The gingiva around molar teeth was excised from the maxilla of 2-month-old WT and *Itgb6*$^{-/-}$ mice under a surgical microscope. Gingival tissues from three mice were pooled together as one biological replicate. The samples were ground over dry ice using a mortar and pestle, followed by extraction of total RNA using NucleoSpin RNA II kit and RT-qPCR to determine the differences in gene expression between WT and *Itgb6*$^{-/-}$ mice. Total of 18 WT and *Itgb6*$^{-/-}$ mice were used in the study per genotype (six biological replicates). PCR primer sequences are listed in Supplementary Table S**2.**

### Immunohistochemistry of mouse gingiva

The gingiva around molar teeth was excised from the maxilla of 9-month-old WT and *Itgb6*$^{-/-}$ mice under a surgical microscope and processed for frozen sections and immunohistochemistry as described for the human tissue. Primary antibodies used for immunostaining were: β1 integrin (rat; mAb13; a kind gift from Dr. Kenneth Yamada, National Institutes of Health, Bethesda, MD, USA), α6 integrin (rat; mAb1378; Chemicon) and αvβ6 integrin (rabbit; β6B1). Primary antibodies were omitted from the negative control stainings. To highlight tissue structure, comparable sections were stained with hematoxylin and eosin, as described.

### Culture of mouse macrophages

The murine macrophage-like cell line RAW 264.7 (ATCC) was used for confirming receptor agonist activity. Cells were cultured as periviously decribed[1] and seeded into plates at $3\times10^4$ cells/cm$^2$ for 48 h in their complete growth medium for the experiments. The cells were then rinsed once with PBS, changed to FBS-free medium and treated with receptor agonists or left untreated for 24 h. RNA isolation and RT-qPCR were performed as described.

### *P. gingivalis* and *T. denticola* bacterial extracts

*P. gingivalis* (ATCC 33277) and *T. denticola* (ATCC 35405) were cultured as previously described[2]. The bacterial extracts were produced and heat-or proteinase K-treated as described for biofilm. The bacterial extracts were tested at concentrations 3-60 μg protein/ml and used in the experiments at concentration of 30 μg protein/ml (maximal efficacy without cytotoxity).

## Genomic PCR of bacterial biofilms

Genomic PCR was used to determine the presence of total oral mycoplasma in the bacterial biofilms and to estimate the relative proportion of these bacteria in them using mycoplasma-specific and universal bacterial primers for 16S ribosomal RNA. The bacterial samples were boiled for 15 min. PCR amplification was performed with the CFX96 system (Bio-Rad; program for *Mycoplasma* gene: 5 min at 95°C, followed by 40 cycles of 30 s at 94°C, 1 min at 55°C and 1 min at 72°C; program for universal bacterial gene: 5 min at 95°C, followed by 20 cycles of 30 s at 94°C, 1 min at 59°C and 1 min at 72°C). The sequences of Mycoplasma primers were: Forward: 5'-ACA CCA TGG GAG CT GGT AAT-3'; Reverse: 5'-CTT CTT CGA CTT TCA GA-CCC AAG -3'. The universal bacterial primer sequences were: Forward: 5'-CAD ACT CCT ACG GGA GGC-3'; Reverse: 5'-ATC CTG TTT GMT MCC CVC RC-3' (M=A/C, R=A/G, V=A/G/C). The PCR products were resolved by agarose gel electrophoresis (1% gel) and the images captured under UV light. ImageJ software was used for the quantification of blots.

# References


1 Barth, K. A., Waterfield, J. D. & Brunette, D. M. The effect of surface roughness on RAW 264.7 macrophage phenotype. J Biomed Mater Res A 101, 2679-2688, doi:10.1002/jbm.a.34562 (2013).

2 Bodet, C., Chandad, F. & Grenier, D. Inflammatory responses of a macrophage/epithelial cell co-culture model to mono and mixed infections with Porphyromonas gingivalis, Treponema denticola, and Tannerella forsythia. Microbes Infect 8, 27-35, doi:10.1016/j.micinf.2005.05.015 (2006).


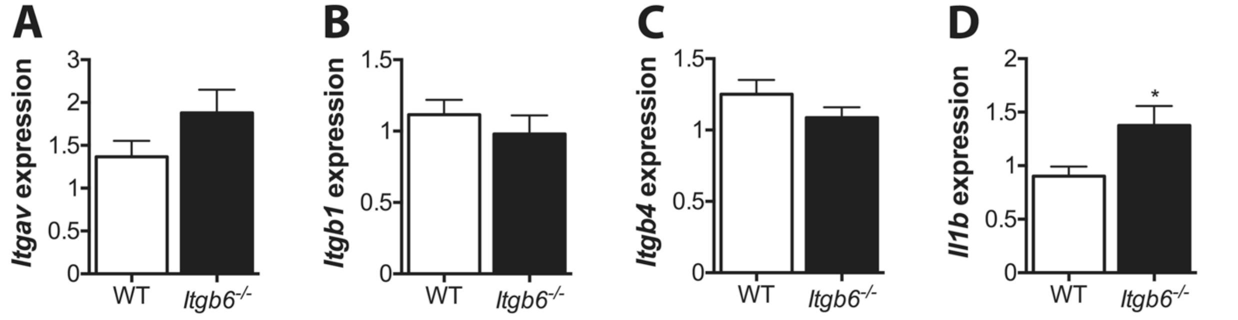


**Supplementary Figure S1. Integrin and IL-1β gene expression in WT and *Itgb6$^{-/-}$* mouse gingiva**

**A-D,** RNA from WT and *Itgb6-/-* mouse gingiva was extracted and analyzed for *Itgav* (A), *Itgb1* (B), *Itgb4* (C) and *Il1b* (D) expression by RT-qPCR. Gingival tissue from three animals was pooled as one biological replicate. Mean ± SEM of total of six replicates (18 mice) per group is presented. *, $p<0.05$.

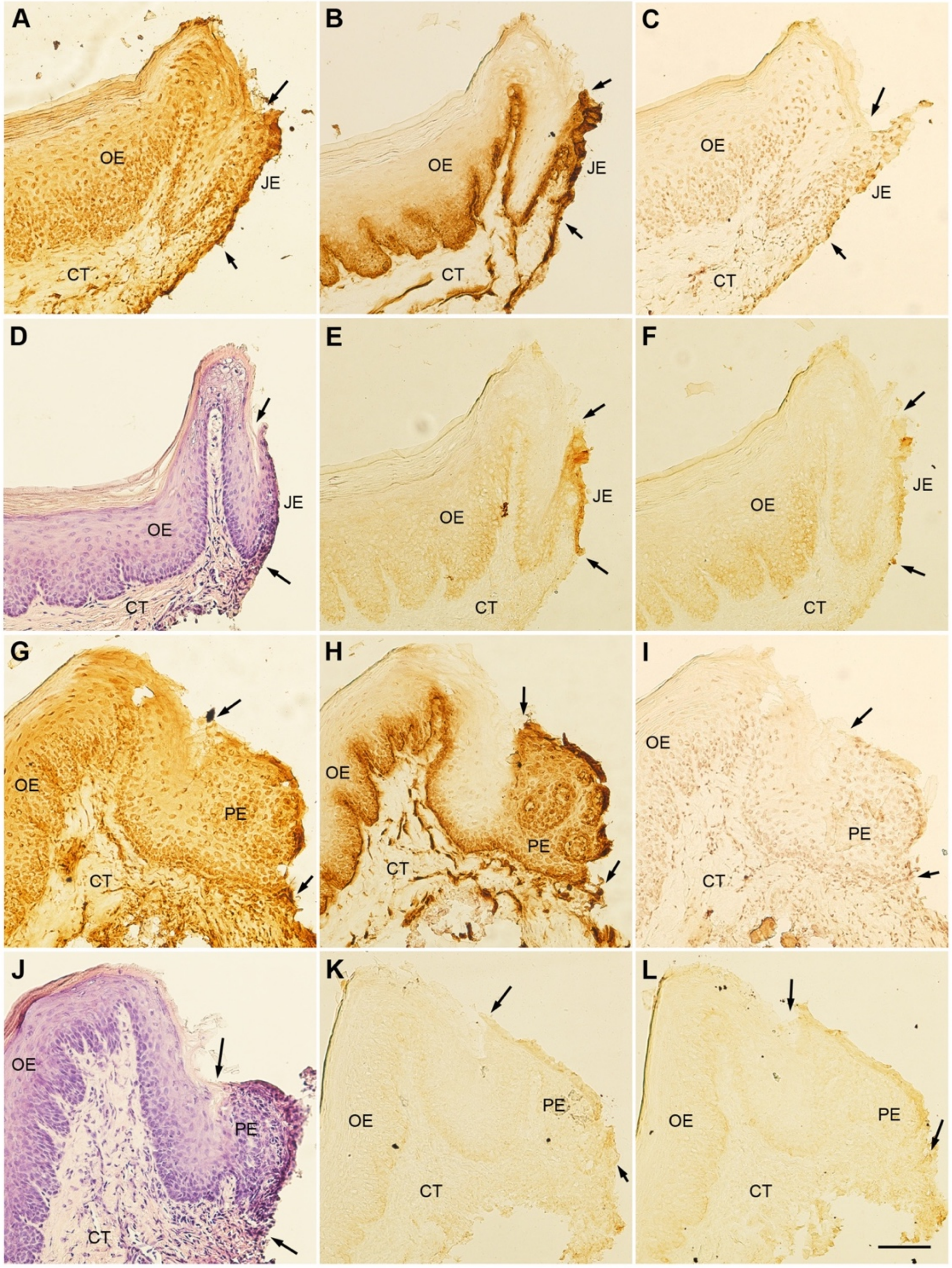


**Supplementary Figure S2. Immunolocalization of integrins in WT and *Itgb6*$^{-/-}$ mouse gingiva**

Immunolocalization of β1 (A, G), α6 (B, H) and αvβ6 integrins (E, K) in WT (E-F) and *Itgb6*$^{-/-}$ (G-L) mouse gingiva. Species-appropriate negative controls were performed with secondary antibodies only: anti-rat (C, I) and anti-rabbit (F, L). Comparable hematoxylin-eosin-stained sections of WT (D) and *Itgb6*$^{-/-}$ (J) mouse gingiva highlight the tissue structure. OE, Oral epithelium; CT, connective tissue; JE, junctional epithelium; PE, pocket epithelium. Arrows point the most coronal and apical aspects of the JE/PE. Bar is 50 μm.

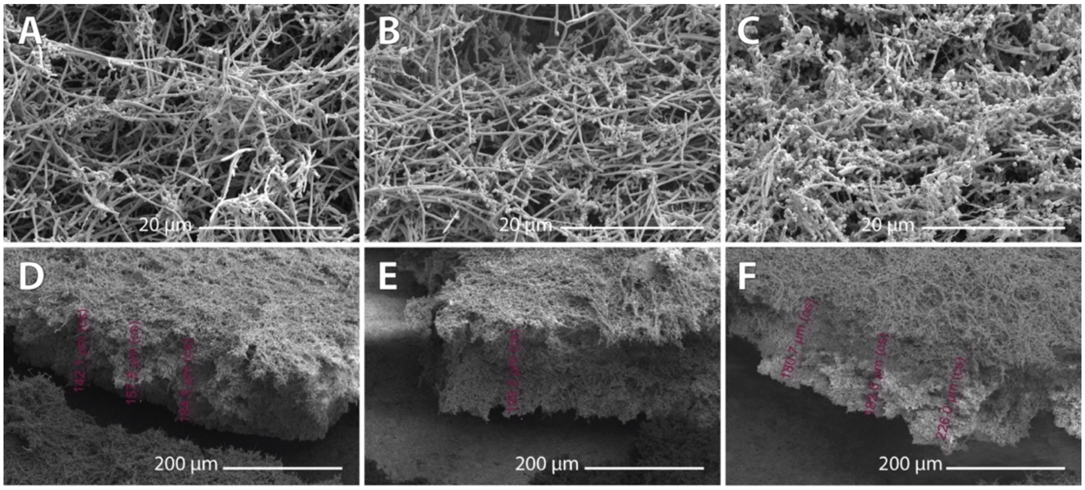


**Supplementary Figure S3. Structure of oral bacterial biofilm from three donors.**

**A-F,** The multi-species oral bacterial biofilms from three different donors were cultured for three weeks for SEM micrographs. (A-C) scale bar = 20 µm; (D-F) scale bar = 200 µm. D-F, Cross section (cs) SEM of oral bacterial biofilms reflects the thickness of biofilms of A-C, respectively.

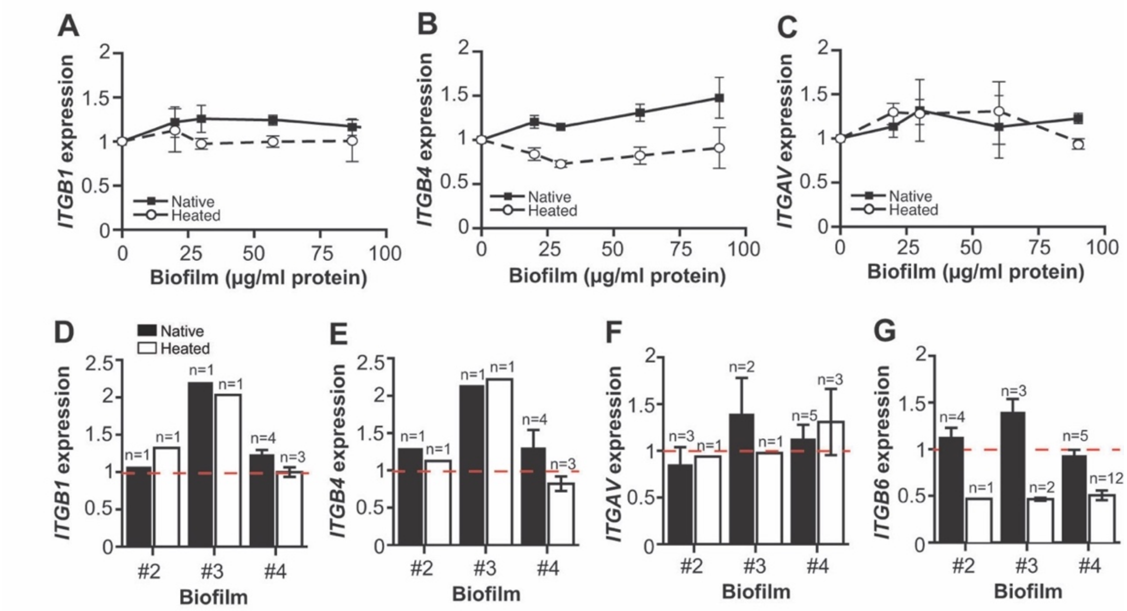


**Supplementary Figure S4. Integrin gene expression in biofilm-treated GECs.**

**A-C,** GECs were exposed to various concentrations of native or heated oral biofilm extract (#4 biofilm; 0-90 µg protein/ml) for 32 h. RT-qPCR was then performed to assess expression of *ITGB1* (A), *ITGB4* (B) and *ITGAV* (C). Mean ± SEM is presented, n=3-5. **D-G,** GECs were treated with either native or heated biofilm extract from three different donors (60 µg biofilm protein/ml) for 32 h and analyzed for *ITGB1* (D), *ITGB4* (E), *ITGAV* (F) and *ITGB6* (G) expression by RT-qPCR. Numbers of experimental repeats are indicated in the Figure. Mean ± SEM is presented. Dashed line indicates the expression level in the untreated control cells.

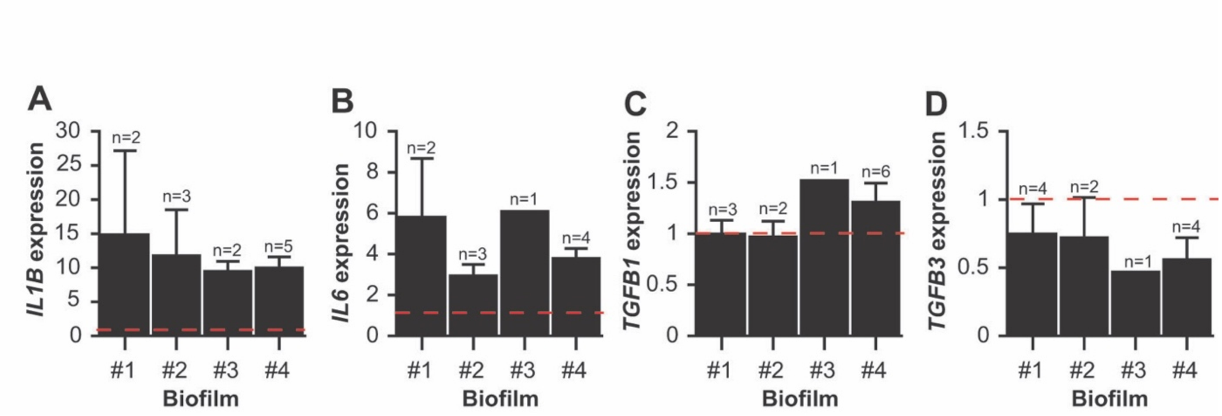


**Supplementary Figure S5. Cytokine gene regulation by four biofilms.**

**A-D,** GECs were treated with either native or heated biofilm extract from four different donors (60 µg protein/ml) for 32 h and analyzed for *IL1B* (A), *IL6* (B), *TGFB1* (C) and *TGFB3* (D) expression by RT-qPCR. Numbers of experimental repeats are indicated in the Figure. Mean ± SEM is presented. Dashed line indicates the expression level in the untreated control cells.

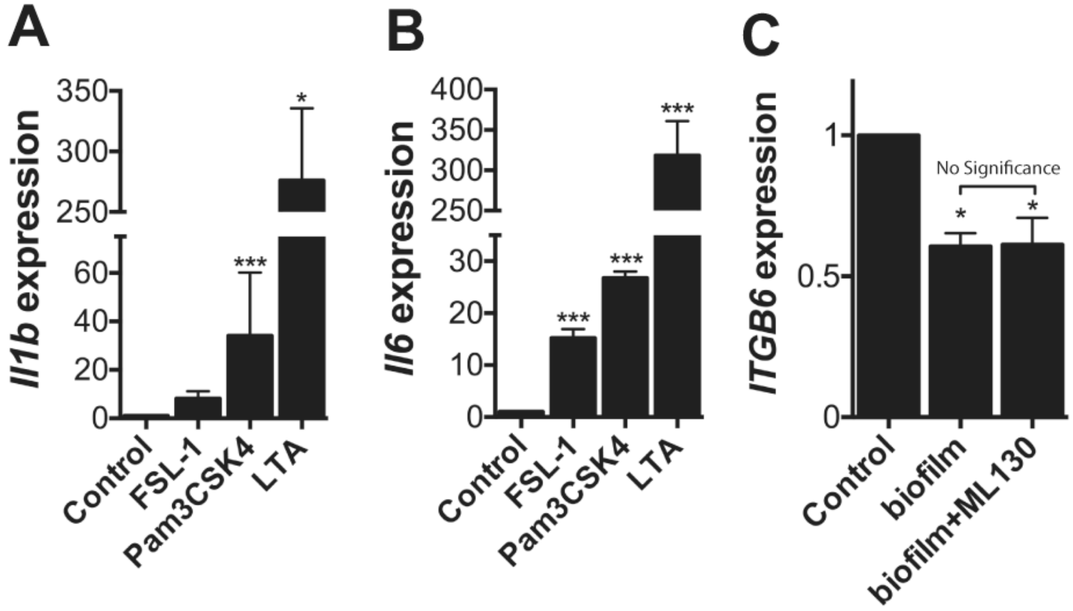


**Supplementary Figure S6. Effects of TLR ligands on cytokine expression in RAW 264.7 macrophage cell line and NOD1 inhibitor on *ITGB6* downregulation in GECs.**

**A and B,** RAW 264.7 cells were treated with FSL-1 (100 ng/ml), Pam3CSK4 (300 ng/ml) or LTA (2 µg/ml) or left untreated for 24 h, and their effect on *Il1b* (A) and *Il6* expression (B) was analyzed by RT-qPCR. Mean ± SEM is presented (n=3). **C,** GECs were pre-treated with NOD1 inhibitor ML130 for 1 h or left untreated, followed by addition of heated biofilm extract (60 µg protein/ml) for 32 h. The samples were analysed by RT-qPCR for *ITGB6* expression. Mean ± SEM is presented (n=4). *, $p<0.05$; ***, $p<0.001$.

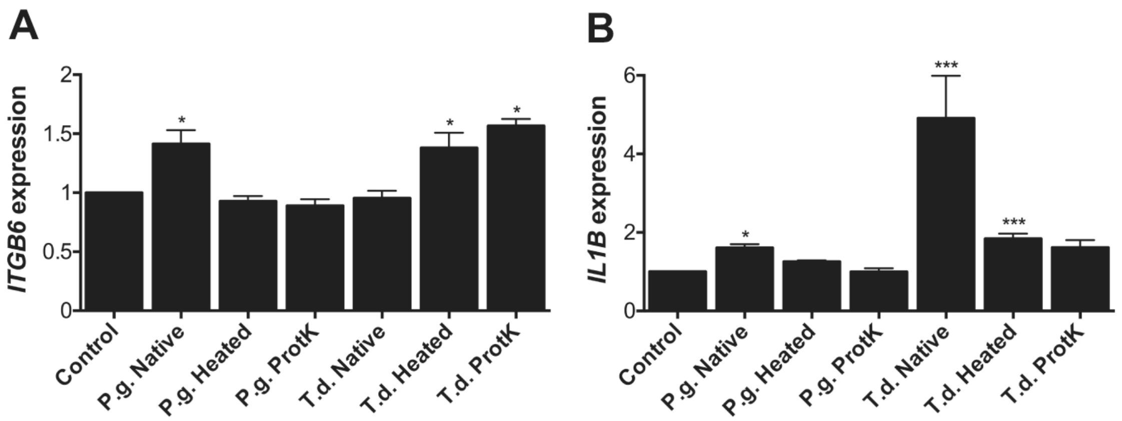


**Supplementary Figure S7. The effects of bacterial extracts of periodontal pathogens *P. gingivalis* and *T. denticola* on *ITGB6* expression in GECs.**

**A and B,** GECs were treated with native, heated, or proteinase K-digested *P. gingivalis* and *T. denticola* extracts (30 µg protein/ml) for 32 h. *ITGB6* and *IL1B* expression was analyzed by RT-qPCR. Mean ± SEM is presented (n=3). *, p<0.05; ***, p<0.001.

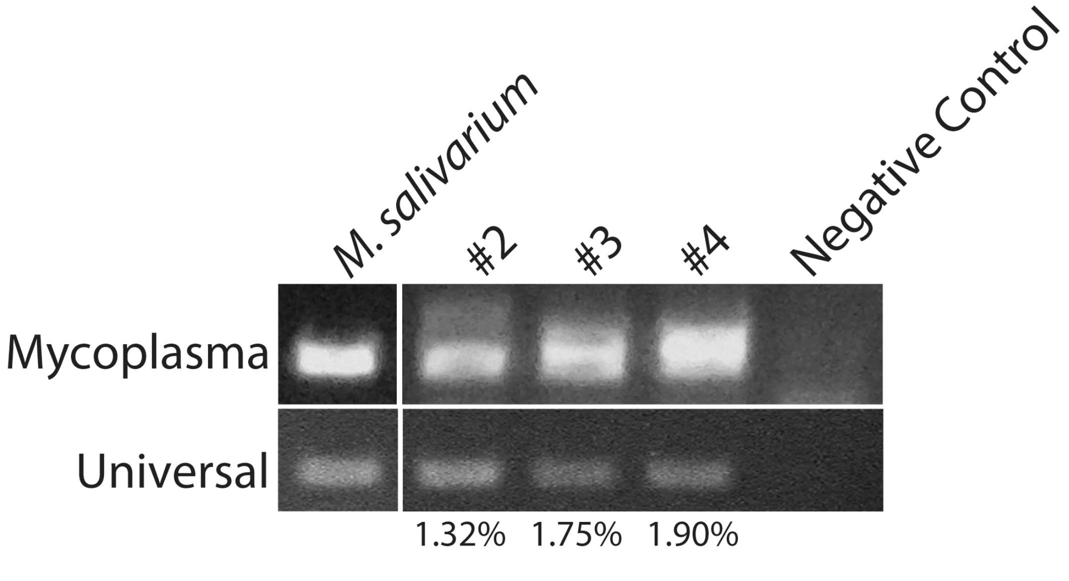


**Supplementary Figure S8. Agarose gel electrophoresis of oral bacterial biofilm PCR products.**

Genomic PCR was performed for oral bacterial biofilms #2, #3 and #4 with mycoplasma-specific and universal bacterial primers (16S ribosomal RNA gene). The biofilm samples were diluted 1:100 for universal bacterial PCR analyses. Relative to Figure 5, *M. salivarium* was diluted 1:10 and used as a positive control for both of mycoplasma and universal bacterial PCR analysis, whereas water was used as a negative control. Agarose gel electrophoresis images were quantitated using ImageJ software, and the ratios of genomic copies for mycoplasma relative to universal genes were calculated and expressed as per cent of total genomic copies.

| Gene | Cq Value | Relative expression |
|---|---|---|
| *Il1b* | 24≤Cq≤27 | Upregulation |
| *Il2* | 35≤Cq≤38 | Low expression |
| *Il6* | 31≤Cq≤33 | Low expression |
| *Il8* | 37≤Cq≤39 | Low expression |
| *Il10* | 30≤Cq≤33 | Low expression |
| *Il17a* | 32≤Cq≤34 | Low expression |
| *Il22* | 35≤Cq≤38 | Low expression |
| *Il23* | 27≤Cq≤39 | Low expression |
| *Tnfa* | 27≤Cq≤29 | No change |

**Supplementary Table S1. Cytokine gene expression in WT and *Itgb6*$^{-/-}$ mouse gingiva.**

| Gene | Cq Value | Relative expression |
|---|---|---|
| *IL1A* | 28≤Cq≤35 | Low expression |
| *IL1B* | 23≤Cq≤27 | Upregulation |
| *IL6* | 24≤Cq≤29 | Upregulation |
| *IL7* | 31≤Cq≤34 | Low expression |
| *IL8* | 30≤Cq≤38 | Low expression |
| *IL10* | 35≤Cq≤38 | Low expression |
| *IL12A* | 29≤Cq≤31 | No change |
| *IL15* | 33≤Cq≤37 | Low expression |
| *IL18* | 27≤Cq≤29 | No change |
| *IL20* | 31≤Cq≤33 | Low expression |
| *IL24* | 33≤Cq≤41 | Low expression |
| *TNFA* | 27≤Cqt≤29 | No change |

**Supplementary Table S2. Cytokine gene expression in biofilm-treated GECs relative to non-treated cells.**

| Gene | GenBank accession number (mRNA) | Forward primer | Reverse primer |
|---|---|---|---|
| ***Homo sapiens*** | | | |
| *TGFB1* | NM_000660 | CAACGAAATCTATGACAAGTTCAAGCAG | CTTCTCGGAGCTCTGATGTG |
| *TGFB3* | NM_003239 | ACACCAATTACTGCTTCCGCAA | GCCTAGATCCTGTCGGAAGTC |
| *ITGAV* | NM_002210 | GAGGAAAGAGTGCAATCTTGTA | GAAGCAGACGACTTCAGAGA |
| *ITGB1* | NM_002211 | ATGCCAAATCATGTGGAGAATG | GGCTTCTAAATCATCACATCGTG |
| *ITGB4* | NM_000213 | CGTGTGAGGAATGCAACTTCAAGG | ACCACCTCCTCGGCTCT |
| *ITGB6* | NM_000888 | AATTGCCAACCCTTGCAGTAG | AATGTGCTTGAATCCAAATGTAG |
| *IL1B* | NM_000576 | CAGTGAAATGATGGCTTATT | CTTCATGTTTAGGGCCA |
| *IL6* | NM_000600 | CAACCTGAACCTTCCAAAGATG | TCTGGCTTGTTCCTCACTAC |
| *IL7* | NM_001199888 | ACTGAATGACTTGTGTTTCCTAAAG | ATGCAGCTAAAGTTCGTGTTTC |
| *IL8* | NM_000584 | CAGAGGGTTGTGGAGAAGTT | GCTTGAAGTTTCACTGGCA |
| *IL10* | NM_000572 | AGAACCTGAAGACCCTCAG | CTTATTAAAGGCATTCTTCACCT |
| *IL12A* | NM_000882 | TCAAGCTCTGCATACTTCTTCAT | ATGACAACGGTTTGGAGGGA |
| *IL15* | NM_172175 | GCAATGAAGTGCTTTCTCTTGG | TCCTCACATTCTTTGCATCCAG |
| *IL18* | NM_001243211 | TGTAACTATCTCTGTGAAGTGTGAG | TCCTGGGACACTTCTCTGAA |
| *IL20* | NM_018724 | GACATCAGAATCTTAAGGAGGAC | ATACCCTGTCCAGATAGAGTC |
| *IL24* | NM_ 006850 | GAGATGTTTTCCATCAGAGACA | CAGGTCAGAAGAATGTCCAC |
| *TNFa* | NM_000594 | GACCTCTCTCTAATCAGCC | TTGAGGGTTTGCTACAACA |
| *MKI67* | NM_001145966 | TTCGGAAGCAAATCTGATTGT | TTGTTCATTGACCTTTGAGGA |
| *TLR1* | NM_003263 | CTGTATCTGTATCAAGATGATCTG | TCTGATCTGAAGTATTAACATGAAG |
| *TLR2* | NM_003264 | TGACTCCATTGAAAAGAGCCA | AAGACGGAAATGGGAGAAGTC |
| *TLR3* | NM_003265 | AGCCTTACAGAGAAGCTATG | TAGGAAAGATCGAGCATAGTG |
| *TLR4* | NM_003266 | AACCTCCCCTTCTCAACCA | GTTCTGGGAAACTGAAGAAGCTA |
| *TLR5* | NM_003268 | GACTTTGCCCATCAATACACAGG | TCTCCAGGTTCGGACAGC |
| *TLR6* | NM_006068 | AGACCTACCGCTGAAAACC | CAAGTAGCTGGATTCTGTTATGG |
| *TLR7* | NM_016562 | CTCAAGCTGATCTTGGCA | TAGTCTTCTTCCAAAATGGAATG |
| *TLR8* | NM_138636 | AGGTAAAAGGCTACAGGTCTC | AGCTCATTTATCACCCAGTCA |
| *TLR9* | NM_017442 | AGCACCTTCTTGGCTGTG | TTGGTATGGCTGAGGGACA |
| *TLR10* | NM_001195108 | ACTGGATTTATCCTATAACCTCC | AACTCTCAGTTTGGAGACAG |
| *NOD1* | NM_006092 | CAGGCTTGGAAGAGACAGA | TCTGGCATCATTCTTTGAAGTTAG |
| *NOD2* | NM_001293557 | GAAGGCTGCTTGATCTTGC | CATATACTTCTTGCATGTGGCA |
| *ALG9* | BC009255 | GAATGACCAGAATCTAGAAGAGCCA | TCTCATGGTGTCCAAATCCACTAAA |
| *B2M* | NM_004048 | TGTCTTTCAGCAAGGACTGGTCTTTC | ATGGTTCACACGGCAGGCATA |
| *GAPDH* | NM_002046 | CTTTGTCAAGCTCATTTCCTGGTA | GGCCATGAGGTCCACCA |
| ***Mus musculus*** | | | |
| *Itgb1* | NM_010578 | GCTGGTTCTATTTCACCTATTCA | CAACCACGCCTGCTACAA |
| *Itgb4* | NM_001005608 | CCAGCTGAGACCAATGGCGA | GAGCACCTTCTTCATAGGTCCA |
| *Itgav* | NM_008402 | GTCAGTCGGCAGGCTC | AGATCTTCTTTTGATCACTATTTACAG |
| *Il1b* | NM_008361 | TCTATACCTGTCCTGTGTAATG | ACTCCACTTTGCTCTTGAC |

| | | | |
|---|---|---|---|
| *Il2* | NM_008366 | TGAGCAGGATGGAGAATTACAG | AGAGGTCCAAGTTCATCTTCTAG |
| *Il6* | NM_031168 | TCGGAGGCTTAATTACACATG | GTTTGGTAGCATCCATCATTTC |
| *Il8* | NM_011339 | ACGGACATGGCTGCTCAA | CTGAATACACAGACATCGTAGCTCT |
| *Il10* | NM_010548 | AGCAGGTGAAGAGTGATTTTAATAAG | CATCATGTATGCTTCTATGCAGT |
| *Il17a* | NM_010552 | TGTGAAGGTCAACCTCAAAGTC | ACTGAGCTTCCCAGATCACA |
| *Il22* | NM_016971 | TGACGACCAGAACATCCAGA | AGACGCAAGCATTTCTCAGAG |
| *Il23* | NM_031252 | ATGGCTACCATGATAAGACTAATC | CTGCTCTGGGGTTTGTTTC |
| *Tnfa* | NM_013963 | TGGGACAGTGACCTGGAC | TTC TGA GAC AGA GGC AACCT |
| *Ubc* | NM_019639 | GCCACCGTGAAACAACTC | CCTCCAGGGTGATGGTCTTA |
| *B2m* | NM_009735 | AAGGACTGGTCTTTCTATATCCTG | ACTCTGCAGGCGTATGTATC |
| *Gapdh* | NM_002046 | CTTTGTCAAGCTCATTTCCTGGTA | GGCCATGAGGTCCACCA |

**Supplementary Table S3. Sequences of primers (5'-3') used for RT-qPCR**

# Original western blot and general PCR panels:

Figure 4A

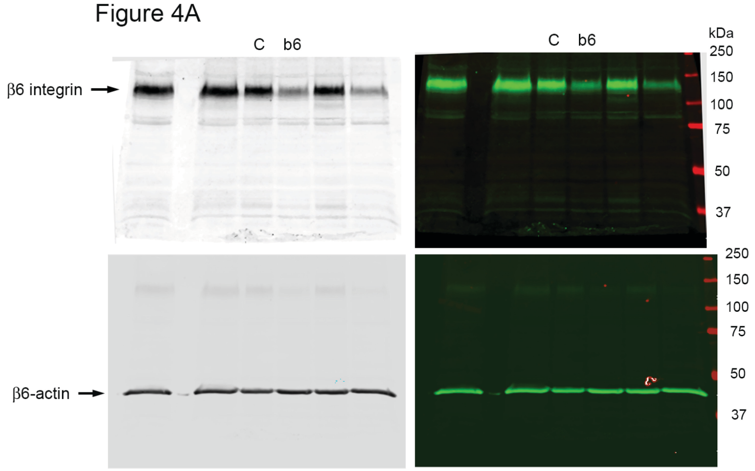


Figure 4E

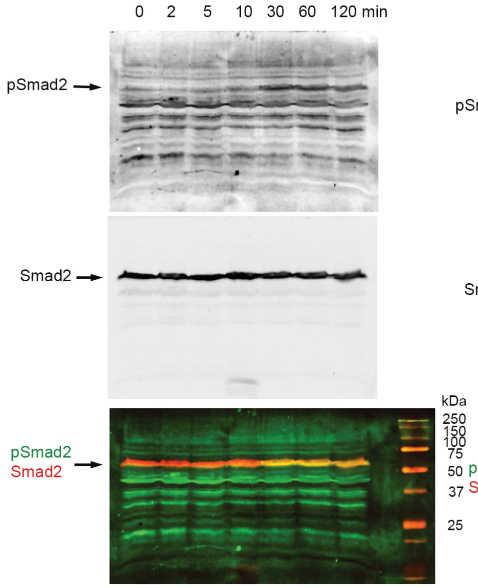


Figure 4F

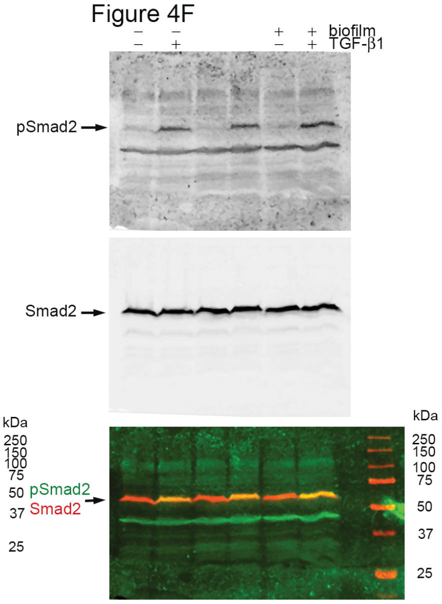


Figure S8 General PCR

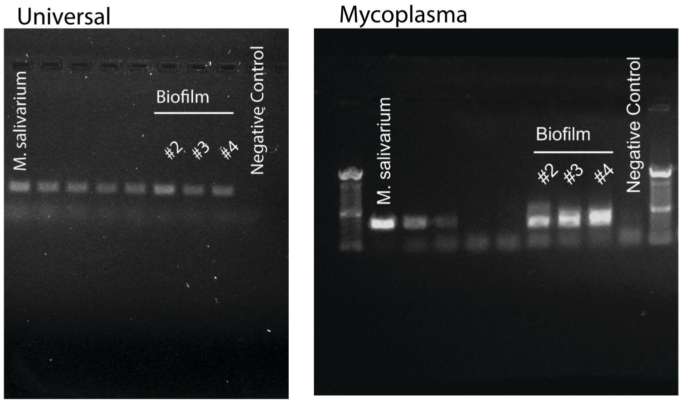

Supplement: Supplementary file 1 — Supplementary material [file 41598_2017_3619_MOESM1_ESM.pdf]
